# Ortholog transfer stress test: Regulatory edge conservation across species, tissues, and evolutionary distance

**DOI:** 10.1371/journal.pone.0347366

**Published:** 2026-08-12

**Authors:** Ihor Kendiukhov

**Affiliations:** Institute of Medical Genetics and Applied Genomics, University of Tübingen, Tübingen, Germany; Chang Gung University, TAIWAN

## Abstract

Researchers routinely take a gene-regulatory network measured in one species—for example, which transcription factor switches which gene on or off in the mouse—and assume the same wiring holds in another species after matching genes by ancestry (orthology). How often this assumption holds quantitatively has not been measured systematically. Here I test it directly. Using single-cell RNA-sequencing of matched organs, I measure, for each transcription factor, how strongly its expression tracks that of every other gene (a co-expression “edge”), compute these edges independently in human and mouse lung, and ask how well they agree. Edges agree strongly when the organ is the same in both species (rank correlation ρ=0.74; the direction of an association—activating versus repressing—matches for 100% of the strongest edges), far beyond chance (*z* = 112 against a permutation null). Agreement is, however, highly uneven: edges of identity-defining factors such as NKX2−1 (lung), ERG (blood vessel), and RUNX3 (lymphocyte) transfer almost perfectly (ρ>0.80), whereas edges of signal-activated factors such as HIF1A, STAT1, and CTNNB1 barely transfer at all (median ρ=0.14 for the signal-responsive group). Strikingly, changing the organ within one species disrupts edges *more* than changing the species within one organ: 76% of factors are better conserved across species than across tissues. The conserved signal is not merely a by-product of matching cell-type proportions: after statistically removing cell-type identity as a covariate, cross-species agreement remains substantial (ρ=0.50). Extending the comparison to zebrafish using a real developmental cell atlas (430 million years of divergence), edge agreement falls to weakly positive (ρ=0.15−0.19), and—contrary to a simple expectation—the identity-versus-signaling gap is no longer statistically distinguishable at that distance. Whole-protein sequence identity is a poor predictor of edge transfer at mammalian distances (*R*^2^ < 0.01). Finally, benchmarking against a curated database of known regulatory interactions shows that co-expression strength is a weak indicator of direct regulation (area under the ROC curve ≈0.52), so what transfers across species is conserved co-expression *structure*—largely cell-identity programs—rather than validated direct regulatory wiring. Together these results give practical, quantitative guidance on when cross-species transfer of regulatory information is safe and when it is not.

## Introduction

Comparative genomics relies on orthology—ancestry-based gene matching—to carry functional knowledge from one species to another [1–3]. In network biology this is extended to regulatory interactions: if two genes are orthologous and their roles are conserved, the regulatory relationship between them is assumed to transfer. This assumption is built into everyday practice, where transcription factor (TF)–target networks inferred or curated in a model organism are projected onto human genes, or vice versa [4,5].

How well this works *quantitatively*—not whether a network can be transferred, but how faithfully the strength and direction of individual regulatory edges survive the transfer—has not been measured at genome scale. Three assumptions are worth stating plainly, because the rest of this paper tests them. First, that the pairwise co-expression structure of a TF and its candidate targets is preserved across species when both genes are orthologs. Second, that organ (tissue) identity and evolutionary distance affect this preservation roughly uniformly across TFs. Third, that protein sequence conservation, used as a proxy for functional equivalence, predicts which edges transfer. Each of these is individually plausible [6,7] and each, as shown below, is partly or wholly wrong.

The growth of single-cell RNA-sequencing (scRNA-seq) [8,9], including matched organs profiled in multiple species, makes a direct test feasible. Cell atlases such as Tabula Sapiens [10] and the Krasnow mouse lung data [11] let one measure TF–target co-expression at single-cell resolution in the same organ across species, and a whole-organism zebrafish atlas [12] extends the comparison across 430 million years. Curated interaction databases [4,5] and perturbation screens [13–15] provide independent yardsticks for whether co-expression edges correspond to real regulation.

This work addresses one question: **under what conditions, and for which transcription factors, do co-expression-based regulatory edges transfer reliably across species?** I measure edge agreement between human and mouse lung; dissect it by TF, by tissue, and against a permutation null; isolate the contribution of cell-type composition by treating cell type as a covariate; extend the comparison to zebrafish using real single-cell data; test whether protein sequence identity predicts transfer; and benchmark the edges against curated ground truth. The concrete deliverables are (i) a quantitative map of which TF programs transfer and which do not, (ii) the finding that tissue identity constrains regulatory co-expression more than species identity, and (iii) quantitative bounds on what conserved co-expression does and does not imply about direct regulation.

## Materials and methods

### Data sources, versions, and access

All single-cell data are publicly available and were obtained from the CZ CELLxGENE Discover portal (https://cellxgene.cziscience.com) in January 2026, except the zebrafish atlas (Gene Expression Omnibus, accession GSE223922) and curated interaction data (see below). The primary cross-species comparison uses two matched lung datasets:

- **Human lung**: Tabula Sapiens [10], 65,847 cells, 60,606 genes, 34 annotated cell types, mixed Smart-seq2 and 10× Genomics, log-normalized expression, human Ensembl identifiers.
- **Mouse lung**: Krasnow laboratory Smart-seq2 data [11], 9,409 cells, 53,514 genes, 40 cell types, log-normalized.

Two additional human organs from Tabula Sapiens were used to test tissue effects, which I refer to throughout as the *extension tissues*:

- **Human kidney**: 11,376 cells, 13 cell types (82% kidney epithelium).
- **Human immune compartment**: 592,317 cells, 45 cell types; subsampled to 12,000 cells (uniformly at random) for tractability.

*Why lung for the cross-species comparison:* Lung is the organ for which high-quality, deeply annotated scRNA-seq is available in both human and mouse from the same era of technology, with overlapping cell-type coverage. I use the term *matched tissue* to mean the same organ profiled in two species, with cell types mapped to a common Cell Ontology vocabulary (Cell Ontology standardization subsection). The cross-species comparison is restricted to mouse lung because a mouse kidney/immune dataset of comparable quality was not available; this restriction is noted as a limitation.

*Quality control:* Each dataset was filtered following standard practice [16]: cells with fewer than 200 detected genes were removed, and cells in which mitochondrial transcripts made up more than 30% of a cell’s total UMI counts (a marker of dying cells) were excluded.

*Interaction and perturbation data:* Curated human TF–target interactions were taken from TRRUST v2 [5] (8,427 directed interactions after restricting to TFs in this study). Perturbation data came from three published Perturb-seq studies [13–15]; their perturbation modalities differ (CRISPR knockout vs. CRISPR-interference knockdown), which matters for interpretation and is discussed in the validation subsection of the Results.

### Orthology

For the human–mouse comparison, the CELLxGENE datasets provide mouse genes already mapped to human Ensembl identifiers; this yields 53,482 shared one-to-one ortholog identifiers. Orthology is inherently more robust when inferred from phylogenetic gene trees than from a single sequence-identity cutoff; accordingly, the zebrafish orthologs (zebrafish subsection) and all reported sequence statistics were obtained from the Ensembl Compara gene-tree pipeline via the Ensembl REST API (release 116) [17], which infers orthology from phylogenetic gene trees rather than reciprocal best hits alone. For each TF I recorded the ortholog type (one-to-one vs. one-to-many) and the whole-protein percent identity. The REST homology endpoint did not expose per-pair $d_{N}/d_{S}$ for these comparisons, so I rely on percent identity; computing $d_{N}/d_{S}$ would require a separate codon-alignment pipeline and is left to future work.

### Edge scoring

For each TF in each dataset I computed the Spearman rank correlation ρ between the TF’s expression and every other gene’s expression, across individual cells after quality control (self-edges excluded). One ρ value is one *edge*. Spearman correlation was chosen for robustness to the outliers and non-linearity typical of single-cell data [18,19] and because it is directly comparable to published co-expression networks. To limit noise, edges with |ρ|<0.05 in a given species were discarded, leaving 121,002 human-lung and 112,319 mouse-lung edges. For the human dataset, cells were subsampled to 10,000 for tractability; targets were the top 3,000 highly variable genes per dataset (Scanpy, cell_ranger flavour [20]). A TF was retained only if expressed in more than 5% of cells in at least one of the analysed datasets (this is what “expressed in at least one condition” means). Cross-referencing a curated TF catalogue [21] with the expressed orthologs left 61 TFs shared between human and mouse lung; all 61 are listed with their DNA-binding-domain family in S1 Table.

### Cross-species edge matching

An edge (TF,target) in human was matched to the edge with the same ortholog pair in mouse. Because the datasets share an identifier space, matching is exact. The conservation analyses below operate on the 25,876 edges that passed the |ρ|>0.05 threshold in *both* species; this set is what I call the *shared edges*, and “conservation analysis” means measuring how the human and mouse ρ values of these shared edges agree.

### Conservation metrics

I quantified agreement with three complementary measures:

- **Edge-score agreement**: the Spearman correlation between the human and mouse ρ values across shared edges (globally, and separately within each TF’s target set, the “per-TF conservation”).
- **Sign agreement**: the fraction of shared edges with the same sign (activating or repressing) in both species, with 95% binomial confidence intervals.
- **Top-*k* overlap**: the number of edges common to the *k* strongest edges (by |ρ|) of each species, reported as fold enrichment over the overlap expected if the two top-*k* lists were drawn independently from the union of all edges.

Per-TF *p*-values were corrected for multiple testing across the 61 TFs by the Benjamini–Hochberg procedure [22]; corrected *q*-values are reported.

### Null models

Three nulls were used, each described in full.

(1) **Gene-permutation null.** For each TF, the mouse edge scores were randomly permuted *among that TF’s own targets* (1,000 permutations), and the global cross-species Spearman correlation recomputed. This preserves each TF’s identity and each species’ marginal distribution of edge scores, but destroys the specific target-to-target correspondence. It tests whether agreement reflects matched targets rather than TF-level or global structure. The observed agreement is reported as a *z*-score and an empirical *p*-value against this null.
(2) **Cell-type-covariate (partial-correlation) control.** To test whether agreement is merely an artefact of both species containing similar proportions of the same cell types, I recomputed every edge as a *partial* Spearman correlation that removes cell-type identity: each gene’s expression was rank-transformed across cells, the mean rank within each Cell-Ontology cell type was subtracted (centering within cell type), and the residuals were correlated. Cross-species agreement computed on these composition-controlled edges measures conservation that cannot be explained by cell-type proportions. This directly answers the question “why not use cell type as a covariate?”
(3) **Independence baseline for thresholds.** To justify the edge-strength cutoffs, I compared, for several |ρ| thresholds, the observed fraction of edges exceeding the threshold in *both* species against the product of the per-species fractions (the value expected if the two species were independent).

### Edge classification and threshold justification

Each shared edge was assigned to one of four classes, using thresholds calibrated against the nulls above rather than chosen arbitrarily:

- **Conserved**: strong and concordant ($\rho_{\text{human}}\times \rho_{\text{mouse}}>0$ with both magnitudes high; top decile of conservation score).
- **Anti-conserved**: strong but opposite sign in the two species.
- **Fragile**: strong in one species and weak in the other, defined as max(|ρ|)>0.3 and $|\;|\rho_{\text{human}}|-|\rho_{\text{mouse}}|\;|>0.25$.
- **Weak**: all remaining edges.

Although |ρ|≈0.2 would be considered weak in bulk data, in sparse single-cell data it is not: requiring |ρ|≥0.2 in *both* species selects edge pairs 2.3-fold more often than expected under independence, rising to 14-fold at |ρ|≥0.4 (Results), so these are co-occurrences well above chance. I also report a sensitivity sweep showing the main conclusions are stable across inclusion thresholds from 0.05 to 0.3.

### Multi-tissue extension

The same edge-scoring pipeline was applied to human kidney and human immune cells. I computed all within-human cross-tissue comparisons (lung–kidney, lung–immune, kidney–immune) and the two cross-species/cross-tissue comparisons (human kidney or immune vs. mouse lung). For each TF I asked whether it was better conserved across species (human lung vs. mouse lung) or across tissues (human lung vs. human kidney/immune); the fraction favouring species was tested against the 50% expectation (the “equal-distribution null”) with a binomial test.

### TF functional categories

The 61 TFs were grouped by a transparent, literature-based scheme [21]: *lineage-specifying* (*n* = 18; identity TFs such as NKX2−1, ERG, RUNX3), *signal-responsive* (*n* = 15; STAT, HIF, NF-κB, SMAD, Wnt/Notch effectors), *immediate-early/stress* (*n* = 9; AP-1 family, EGR1, ATF3, XBP1), and *ubiquitous/general* (*n* = 17; chromatin and basal factors). I emphasise that the lineage/signaling boundary is a continuum, not a hard line—many identity factors act downstream of signals—which is precisely why immediate-early/stress factors are kept as a separate, explicitly intermediate class. Category differences were tested by a one-sided Mann–Whitney *U* test (alternative: lineage-specifying > signal-responsive), and the key contrast was checked for robustness by randomly reassigning 20% of lineage/signaling labels 200 times and re-testing.

### Validation against curated and perturbation data

To ask whether co-expression edges correspond to real regulation, I benchmarked them against TRRUST v2 [5]. Restricting to edges whose TF is curated in TRRUST, I labelled each edge as a known interaction or not and measured how well edge strength and cross-species conservation discriminate known interactions, by area under the ROC curve (AUROC), and whether conserved edges are enriched for known interactions (Fisher’s exact test). Separately, I cross-referenced edges with the three Perturb-seq datasets, interpreting results cautiously given the cell-type mismatch between those screens (K562, T cells) and lung.

### Protein sequence identity

For each TF I correlated the real Ensembl whole-protein percent identity (human–mouse and human–zebrafish) with per-TF cross-species conservation. I also tested whether sequence identity improves a logistic classifier of conserved vs. non-conserved edges beyond edge strength. Whole-protein identity is reported; DNA-binding-domain family is given for context, and the limitation that domain-restricted identity might be more informative is stated explicitly.

### Zebrafish and three-species analysis

To extend beyond mammals I used real zebrafish (*Danio rerio*) single-cell data from Daniocell, a whole-organism developmental atlas [12] (GEO GSE223922; GRCz11 / Lawson v4.3.2 annotation; 489,686 cells, subsampled to 50,000 for tractability, log-normalized). **Zebrafish has no lung**; rather than invent a “lung-equivalent”, I computed edges across the whole atlas, which contains the relevant endothelial/vascular, hematopoietic/immune, and epithelial (including pronephric kidney) lineages, and compared them to human and mouse lung edges on shared ortholog pairs. All 61 human TFs had zebrafish orthologs; 42 were expressed in more than 5% of cells. The teleost-specific whole-genome duplication produces co-orthologs (ohnologs) for many genes (15 of the 61 TFs had one-to-many zebrafish orthology by Ensembl gene trees, and one further TF was many-to-many). Rather than discard these, I analysed the data two ways: using the single best-expressed ohnolog, and averaging over all ohnologs; results were essentially identical (Results), so duplication does not drive the conclusions. Zebrafish diverged from the human *and* the mouse lineage at the same node (the actinopterygian–sarcopterygian split, ≈430 million years ago), so the human–zebrafish and mouse–zebrafish divergence times are equal. With only two distinct divergence times (90 and 430 Mya) I do not fit a precise decay half-life; I report the conservation at each distance directly.

### Cell Ontology standardization

Human and mouse cell types were mapped to Cell Ontology (CL) terms [23]—this is the “matching annotation standard” referred to elsewhere—identifying 19 shared cell types across epithelial, endothelial, immune, and stromal compartments. Cell-type composition was compared by Jensen–Shannon divergence, and conservation was recomputed with edges weighted by the harmonic mean of cell-type fractions, complementing the stronger covariate control of the null-models subsection.

## Results

### Co-expression edges agree strongly within matched tissue

**Fig 1:**
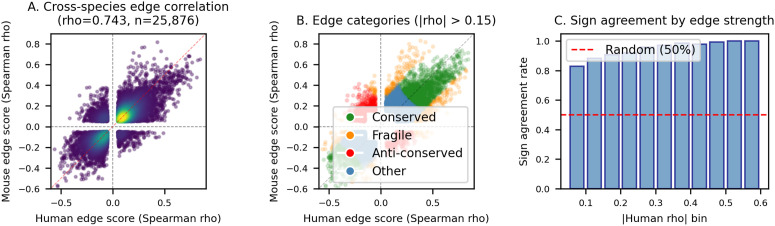
Global conservation of TF–target edges in matched tissue. Human (x) vs. mouse (y) Spearman edge scores for the 25,876 shared lung edges; global ρ=0.743. Sign agreement is 88.6% overall and 100% for strong edges.

**Fig 2:**
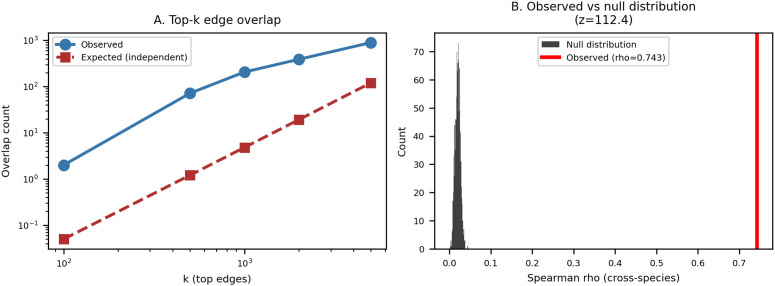
Top-*k* overlap enrichment and the gene-permutation null. Top-*k* overlap enrichment (Table 2) and the gene-permutation null distribution against which the observed ρ=0.743 gives z = 112.

Across the 25,876 shared human–mouse lung edges, human and mouse edge scores agreed strongly (Spearman ρ=0.743, Pearson *r* = 0.777; Fig 1). This far exceeds the gene-permutation null (null mean ρ=0.020±0.006; *z* = 112, empirical *p* < 10^−3^ in 1,000 permutations; Fig 2). Sign agreement rose from 88.6% over all edges to 99.3% for edges with |ρ|>0.2 in both species and 100% for |ρ|>0.4 (Table 1), so the direction of strong associations is essentially deterministic across species. The edge-strength thresholds are justified by the independence baseline: the fraction of edges strong in *both* species exceeds the independence expectation 2.3-fold at |ρ|≥0.2 and 14-fold at |ρ|≥0.4, confirming that such co-occurrences are not chance. After Benjamini–Hochberg correction, 51 of 61 TFs showed significant cross-species conservation at *q* < 0.05.

**Table 1:** Sign agreement increases with edge strength (95% binomial CIs).

Threshold |ρ|≥	*n* edges	Sign agreement	95% CI	*p* vs. 50%
0.0	25,876	88.6%	(88.2, 89.0)	<10^−300^
0.1	11,941	95.1%	(94.7, 95.5)	<10^−300^
0.2	3,250	99.3%	(99.0, 99.6)	<10^−300^
0.3	986	99.9%	(99.4, 100)	$\sim 10^{-294}$
0.4	395	100%	(99.1, 100)	$\sim 10^{-119}$

### The strongest edges overlap far beyond chance

**Fig 3:**
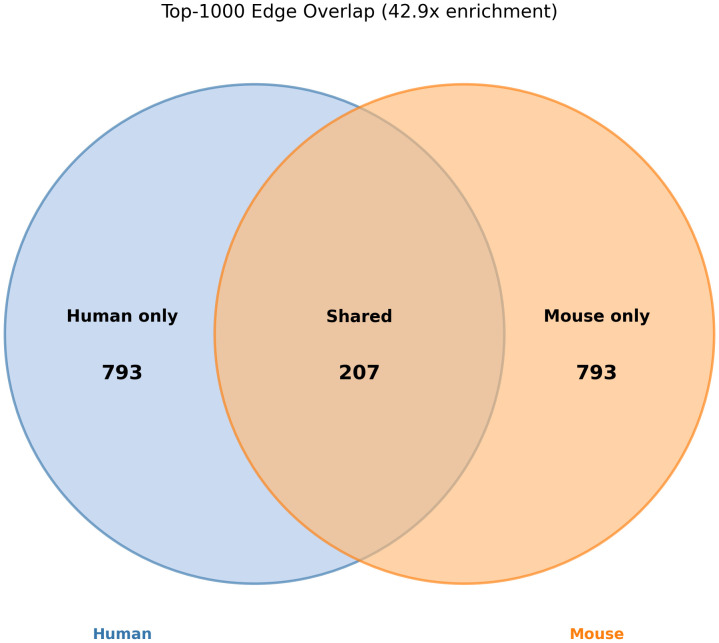
Overlap of the top-1000 strongest edges. Overlap of the top-1000 strongest edges between human and mouse lung, illustrating the enrichment quantified in Table 2.

The *k* strongest edges in each species overlapped well above the independence expectation at every *k* tested (Table 2; the overlap of the top-1000 strongest edges is shown as a Venn diagram in Fig 3), peaking at 60-fold for *k* = 500 and remaining 7-fold enriched at *k* = 5,000. The decline at large *k* shows that the most confidently inferred edges are the most reproducible.

**Table 2:** Top-*k* edge overlap (union universe = 207,445 edges).

*k*	Observed overlap	Expected (independent)	Fold enrichment
100	2	0.05	41.5×
500	72	1.21	59.7×
1000	207	4.82	42.9×
2000	390	19.28	20.2×
5000	897	120.5	7.4×

### Conservation varies enormously across transcription factors

**Fig 4:**
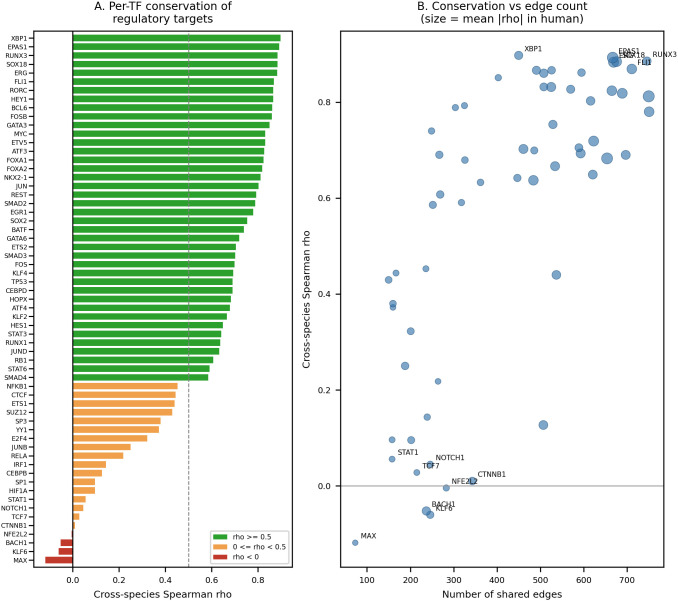
Per-TF conservation ranked across the 61 shared TFs. Identity-specifying factors (top: XBP1, EPAS1, ERG, NKX2−1) versus signal-responsive factors (bottom: STAT1, HIF1A, CTNNB1, MAX). Values are real per-TF Spearman correlations.

**Fig 5:**
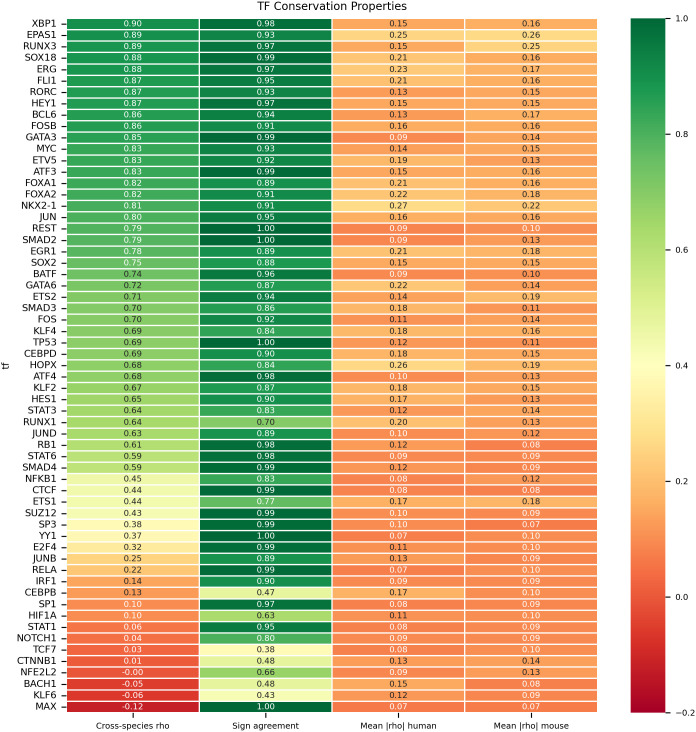
Per-TF conservation properties. Heatmap of per-TF conservation properties (edge count, mean magnitude, sign agreement, cross-species ρ) for the 61 shared TFs.

Per-TF conservation ranged from ρ=−0.12 to ρ=0.90 (median 0.68; Fig 4); the full per-TF property matrix—edge count, mean magnitude, sign agreement, and cross-species ρ for all 61 shared TFs—is summarized as a heatmap in Fig 5. The best-conserved TFs were identity-specifying factors and a small set of conserved stress regulators: XBP1 (0.90), EPAS1 (0.89), RUNX3 (0.89), SOX18 (0.88), ERG (0.88), FLI1 (0.87), GATA3 (0.85), FOXA1/2 (0.82), NKX2−1 (0.81). The worst-conserved were signal-responsive or broadly expressed factors: MAX (−0.12), KLF6 (−0.06), BACH1 (−0.05), NFE2L2 (−0.004), CTNNB1 (0.01), TCF7 (0.03), STAT1 (0.06), HIF1A (0.10). The pattern is biologically coherent: factors that define a stable cell identity transfer well, whereas factors whose activity depends on species-divergent external signals do not.

### Conserved edges are dominated by epithelial identity programs

**Fig 6:**
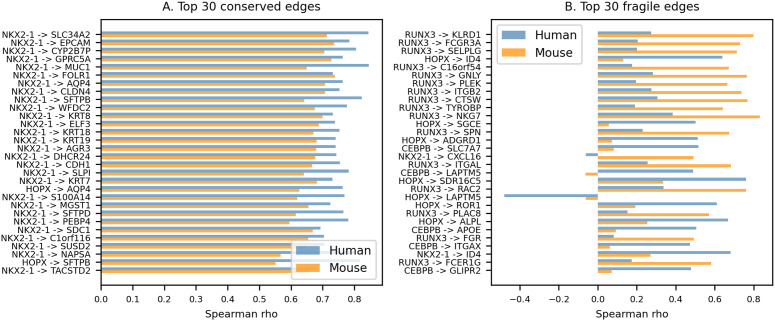
Representative conserved and fragile edges. Representative conserved edges (NKX2−1 epithelial targets, Table 3) and fragile edges (immune programs that differ in cell-type abundance between datasets).

The most conserved edges were overwhelmingly NKX2−1 targets—NKX2−1 is the master regulator of lung epithelial identity (Table 3, Fig 6). Its top conserved targets are core lung-epithelial genes (the ion transporter SLC34A2, the epithelial adhesion molecule EPCAM, the mucin MUC1), the surfactant and cytokeratin programs of which are deeply conserved across mammals.

**Table 3:** Top NKX2−1 conserved targets (real edge scores).

Target	Human ρ	Mouse ρ	Conservation score
SLC34A2	0.844	0.713	0.602
EPCAM	0.784	0.737	0.578
CYP2B7P	0.805	0.706	0.568
GPRC5A	0.763	0.728	0.555
MUC1	0.845	0.650	0.550

### Fragile and anti-conserved edges reflect cell-composition differences

Of the shared edges, 2,362 were conserved, 599 fragile, and 2,535 anti-conserved. Fragile edges (strong in one species, weak in the other) were concentrated in immune programs whose cell types differ in abundance between the datasets—for example RUNX3-driven cytotoxic targets (NKG7, GZMB) that are stronger in mouse, where natural killer cells are more abundant. Importantly, the gene-permutation null produces *more* apparent fragility than the data: 1,887 fragile edges expected by chance versus 599 observed. Real cross-species edges are therefore three-fold *more* concordant in magnitude than random pairing, so fragility is a conservative minority signal driven by genuine compositional and immunological differences, not a noise artefact. Anti-conserved edges were similarly traceable to cell-type composition mismatch (e.g., lymphoid–myeloid axis differences for ETS1).

### Cross-species agreement survives controlling for cell type

**Fig 7:**
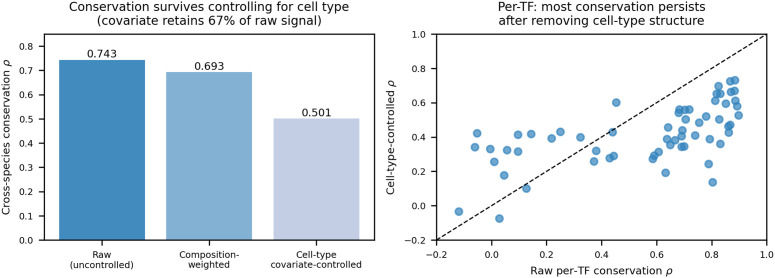
Cell-type composition and its effect on conservation. Cell-type-covariate control reduces global ρ from 0.743 to 0.501 (67% retained); composition weighting reduces it to 0.693. TF divergence classes are shown.

**Fig 8:**
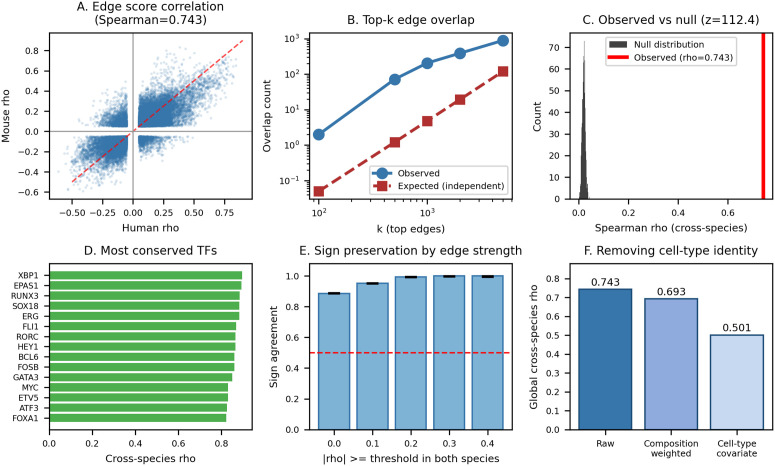
Summary of Phase-1 conservation metrics. Global agreement, sign agreement by edge strength, top-*k* enrichment, and the cell-type-covariate-controlled estimate (ρ=0.50).

A central concern is whether agreement merely reflects matched cell-type proportions. It does not. Recomputing every edge as a partial correlation that removes cell-type identity (null-models subsection) reduced global agreement from ρ=0.743 to ρ=0.501—still strong, retaining 67% of the raw signal (per-TF median fell from 0.68 to 0.41). Thus roughly two-thirds of the conserved co-expression structure is intrinsic to the regulatory relationships and not explained by which cell types are present. The complementary Cell-Ontology composition weighting gave a smaller correction (global ρ from 0.743 to 0.693), bracketing the composition contribution between these two estimates (Fig 7). These Phase-1 conservation metrics—global agreement, sign agreement by edge strength, top-*k* enrichment, and the cell-type-covariate-controlled estimate (ρ=0.50)—are summarized together in Fig 8.

### Tissue identity matters more than species identity

**Fig 9:**
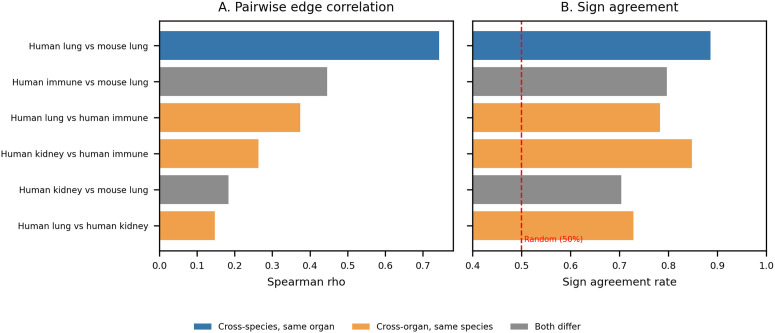
Cross-tissue vs. cross-species agreement. Cross-tissue vs. cross-species agreement (Table 4). Same-organ cross-species agreement greatly exceeds cross-organ same-species agreement.

**Fig 10:**
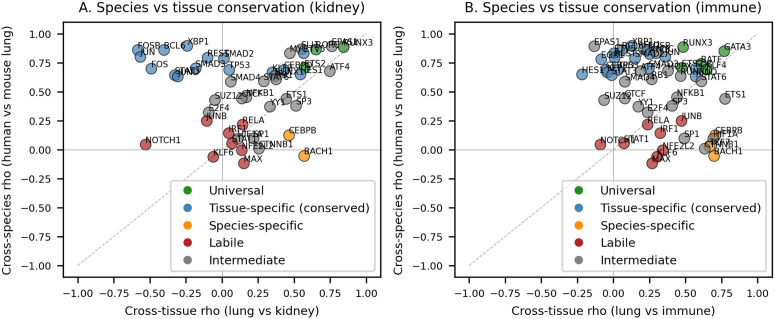
Per-TF cross-species vs. cross-tissue conservation. 76% of TFs (kidney) and 69% (immune) lie on the cross-species-favoured side (binomial *p* < 0.001).

Extending to kidney and immune cells revealed that changing organ disrupts edges more than changing species (Table 4, Figs 9 and 10). Human lung vs. mouse lung agreed at ρ=0.743; but human lung vs. human kidney agreed at only ρ=0.147 and human lung vs. human immune at ρ=0.374, despite identical genes and TFs. Per-TF, 35 of 46 informative TFs (76%) were better conserved across species than across tissues for kidney, and 36 of 52 (69%) for immune (binomial *p* < 0.001 for both). Switching organ within one species is, for most TFs, a larger perturbation to regulatory co-expression than switching species within one organ.

**Table 4:** Cross-tissue agreement is far lower than cross-species agreement.

Comparison	Spearman ρ	*n* shared edges
Human lung vs. mouse lung (cross-species, same organ)	0.743	25,876
Human immune vs. mouse lung (cross-species, cross-organ)	0.445	11,262
Human lung vs. human immune (same species, cross-organ)	0.374	32,536
Human kidney vs. human immune (same species, cross-organ)	0.264	27,148
Human kidney vs. mouse lung (cross-species, cross-organ)	0.184	10,169
Human lung vs. human kidney (same species, cross-organ)	0.147	22,373

### TF functional category predicts transfer, robustly

**Fig 11:**
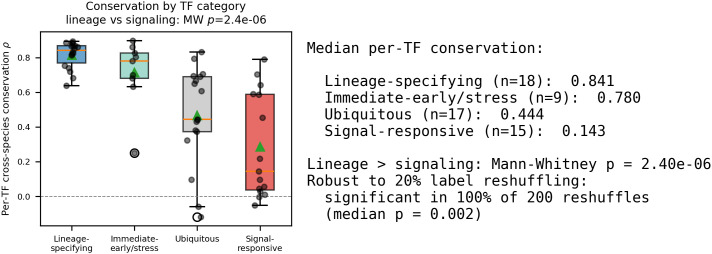
Conservation by TF functional category. Lineage-specifying (median ρ=0.84) ≫ signal-responsive (0.14); immediate-early/stress intermediate-to-high (0.78); ubiquitous intermediate (0.44). Mann–Whitney $p=2.4\times 10^{-6}$, robust to 20% relabelling.

Conservation differed sharply by category (Fig 11): lineage-specifying TFs had median per-TF ρ=0.841 (*n* = 18) versus 0.143 for signal-responsive TFs (*n* = 15); Mann–Whitney $p=2.4\times 10^{-6}$. The immediate-early/stress class was intermediate-to-high (median 0.780, *n* = 9) and the ubiquitous class intermediate (0.444, *n* = 17). Because the lineage/signaling boundary is a continuum, I verified robustness: randomly reassigning 20% of lineage/signaling labels, the contrast remained significant in 100% of 200 reshuffles (median *p* = 0.002). The high conservation of immediate-early factors—which are signal-induced yet identity-stable in their co-expression—illustrates that the meaningful axis is not “signal-responsive vs. not” but how tightly a factor’s targets are tied to cell identity.

### TF divergence classes

**Fig 12:**
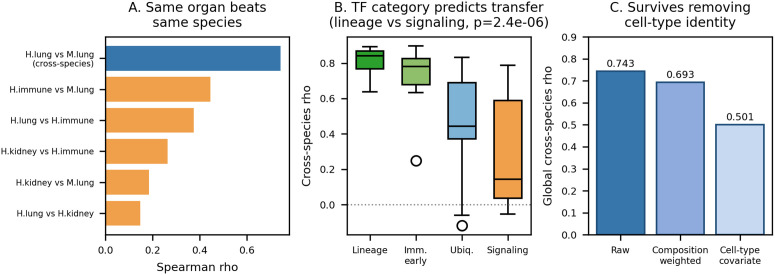
Phase-2 summary. Tissue dominance, category effect, and the cell-type-covariate control.

Combining cross-species and cross-tissue behaviour, TFs fall into four classes (Fig 7): *universal* (conserved across both axes; e.g., RUNX3, GATA3, RORC), *tissue-specific but species-conserved* (the largest class; e.g., NKX2−1, FOXA1/2, XBP1), *species-specific* (e.g., CEBPB, TCF7), and *labile* (poor on both axes; e.g., STAT1, NOTCH1, MAX). Most regulatory programs are tissue-restricted yet evolutionarily deep. The tissue dominance, the category effect, and the cell-type-covariate control are drawn together in the Phase-2 summary (Fig 12).

### Protein sequence identity does not predict transfer at mammalian distances

**Fig 13:**
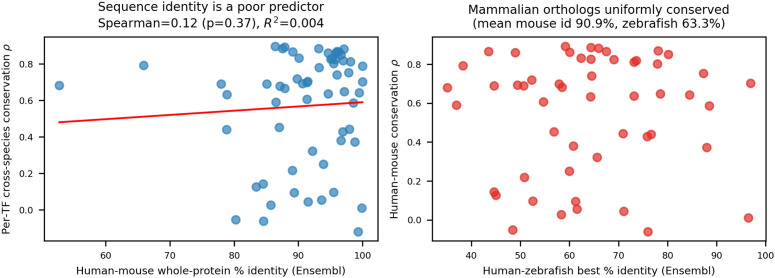
Real Ensembl whole-protein identity vs. per-TF conservation. Human–mouse *R*^2^ < 0.01 (ρ=0.12, *p* = 0.37); identity is a poor predictor at mammalian distances.

Using real Ensembl orthology (release 116), mouse TF orthologs averaged 91.0% whole-protein identity (60 of 61 TFs resolved, all one-to-one) and zebrafish orthologs 63.9% (52 of 61 TFs resolved; 15 one-to-many cases comprising 30 teleost-duplicated ohnolog genes, plus 1 many-to-many case; 9 TFs had no zebrafish ortholog returned by the Ensembl Compara gene-tree pipeline). Sequence identity barely correlated with per-TF conservation: human–mouse Spearman ρ=0.12 (*p* = 0.37, *R*^2^ < 0.01); human–zebrafish ρ=0.05 (*p* = 0.72; Fig 13). The reason is that mammalian TF orthologs are uniformly highly conserved in sequence, leaving little variance to predict regulatory divergence. Edge strength, not sequence identity, dominates any classifier of conserved edges. (Domain-restricted identity might carry more signal and was not exhaustively computed; this is noted as a limitation.)

### Conserved co-expression is weakly tied to direct regulation

**Fig 14:**
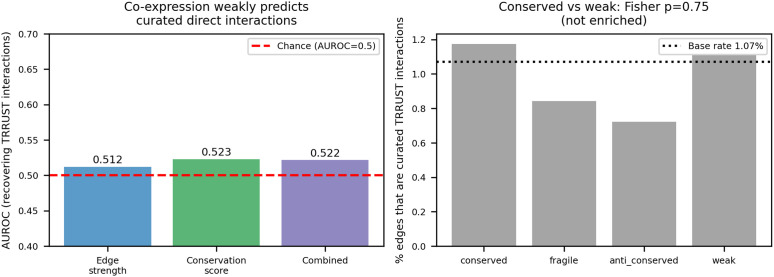
Validation against curated ground truth (TRRUST) and Perturb-seq. Co-expression strength and conservation discriminate curated direct interactions only marginally (AUROC ≈0.52), indicating that conserved co-expression reflects structure more than validated direct regulation.

Benchmarking against TRRUST (8,427 curated interactions; 59 of 61 TFs represented), co-expression edge strength discriminated curated direct interactions only marginally better than chance (AUROC = 0.51), as did the conservation score (AUROC = 0.52), and conserved edges were not significantly enriched for curated interactions over weak edges (1.17% vs. 1.11%; Fisher *p* = 0.75; Fig 14). Direct Perturb-seq overlap was likewise sparse: of six TFs with both edges and perturbation data, only five edges were directly validated, limited by the mismatch between the screens’ cell types (K562, T cells) and lung, and by the difference between knockout and knockdown modalities. The interpretation is important: what transfers conserved across species is co-expression *structure*—largely cell-identity covariation—rather than experimentally validated direct regulatory wiring.

### Three-species comparison with real zebrafish data

**Fig 15:**
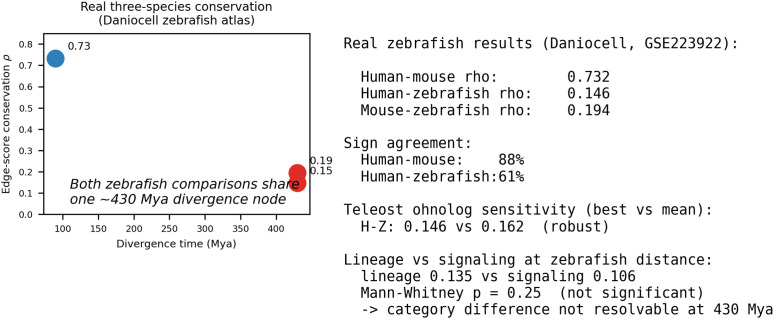
Real three-species conservation using the Daniocell zebrafish atlas. Agreement is strong human–mouse (0.73) and weakly positive at the shared ≈430 Mya zebrafish distance (0.15–0.19). Ohnolog handling (best vs. averaged) does not change the result (Table 5).

Using the Daniocell atlas, I computed real zebrafish edges and compared all three species on 17,792 shared ortholog edges (Table 5, Fig 15). Human–mouse agreement on this set was ρ=0.73. At the much greater zebrafish distance (≈430 Mya from both mammals), agreement fell to weakly positive: human–zebrafish ρ=0.146, mouse–zebrafish ρ=0.194, with sign agreement near 61–64% (versus 88% human–mouse). Consistent with the corrected single divergence node, the two zebrafish comparisons gave similar values. Using the best ohnolog or averaging all ohnologs changed nothing materially (human–zebrafish 0.146 vs. 0.162), confirming that teleost duplication does not drive the result.

**Table 5:** Real three-species conservation (Daniocell zebrafish atlas).

Species pair	Distance (Mya)	Spearman ρ	Sign agreement
Human–mouse	90	0.73	88%
Mouse–zebrafish	≈430	0.19	64%
Human–zebrafish	≈430	0.15	61%

### The lineage advantage is not resolvable at teleost distance

The lineage-vs-signaling difference in zebrafish conservation was *not* statistically significant (lineage median 0.135 vs. signaling 0.106; Mann–Whitney *p* = 0.25). The conclusion is therefore narrow but secure: cross-species conservation is strong within mammals, is clearly category-dependent within mammals (TF functional category subsection), and collapses to weak positive values by teleost distance for all categories, where the category difference is no longer distinguishable at the available sample size.

### TF family × tissue patterns

**Fig 16:**
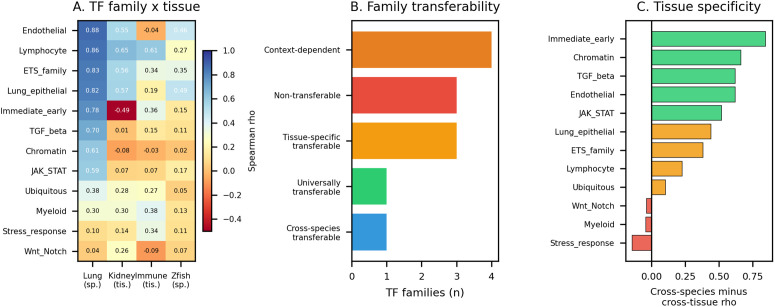
TF family × tissue conservation. ETS factors transfer most broadly across tissues; immediate-early and Wnt/Notch families are the most context-dependent.

Examining conservation across TF families and tissue contexts (Fig 16), ETS-family factors (ERG, FLI1, ETS1/2) were the most broadly transferable across tissues, while immediate-early and Wnt/Notch families were the most context-dependent. These family patterns are consistent with the per-category results and with the known broad, conserved genomic binding of ETS factors [24], but I treat “universally transferable” as a relative description (robust across the tested contexts above null), not an absolute claim.

## Discussion

### What transfers, and what does not

Reading regulatory information from one species into another works well for some things and fails for others, and the results here make the boundary quantitative. Within a matched organ, co-expression edges between orthologous genes agree strongly across human and mouse (ρ=0.74; 100% sign agreement for strong edges), and this holds well above any permutation null and after removing cell-type composition as a covariate (ρ=0.50). The agreement is carried by cell-identity programs: factors such as NKX2−1, ERG, and RUNX3 that define what a cell *is* transfer almost perfectly, because the gene programs that build an epithelial cell, a blood-vessel cell, or a lymphocyte are under strong, shared selection. Factors whose activity is set by external, species-variable signals—hypoxia (HIF1A), interferon (STAT1), Wnt (CTNNB1)—do not transfer.

### Tissue identity constrains regulatory co-expression more than species identity

The most counter-intuitive result is that, for most factors, moving between organs of one species scrambles co-expression edges more than moving between species in one organ. The interpretation is that what a co-expression edge mostly captures is the covariation of genes *within the cell types that make up a tissue*. Because the cellular composition and regulatory context of lung, kidney, and immune tissue differ profoundly, the same TF’s apparent targets differ across organs more than they differ between two mammals’ versions of the same organ. This is why “tissue identity constrains regulatory networks”: the network one infers is, to a large degree, a read-out of the tissue’s cellular makeup, which is far more similar between species (same organ) than between organs (same species).

### Universal versus context-adapted factors

The data separate factors that are functionally universal—conserved across both species and tissues, such as RUNX3 and GATA3—from those adapted to a specific context. The clean version of this distinction (lineage vs. signal-responsive) is statistically strong within mammals (median ρ 0.84 vs. 0.14, robust to relabelling), but the boundary is genuinely a continuum: immediate-early/stress factors are signal-induced yet retain conserved, identity-linked co-expression. The practical message is to weight cross-species confidence by how tightly a factor’s targets are tied to cell identity, not by a binary label.

### Limits of co-expression evidence

Two limits matter for how strongly these results should be read. First, benchmarking against curated interactions shows that conserved co-expression is only weakly predictive of direct regulation (AUROC ≈0.52); conserved edges should be read as conserved *associations*, predominantly cell-identity covariation, not as validated direct TF → target links. Second, at the zebrafish distance the apparent advantage of lineage factors is not statistically resolvable. Stating these limits plainly keeps the focus on the secure findings—strong, category-dependent conservation within mammals, and the primacy of tissue identity.

### Implications for cross-species network transfer

**Fig 17:**
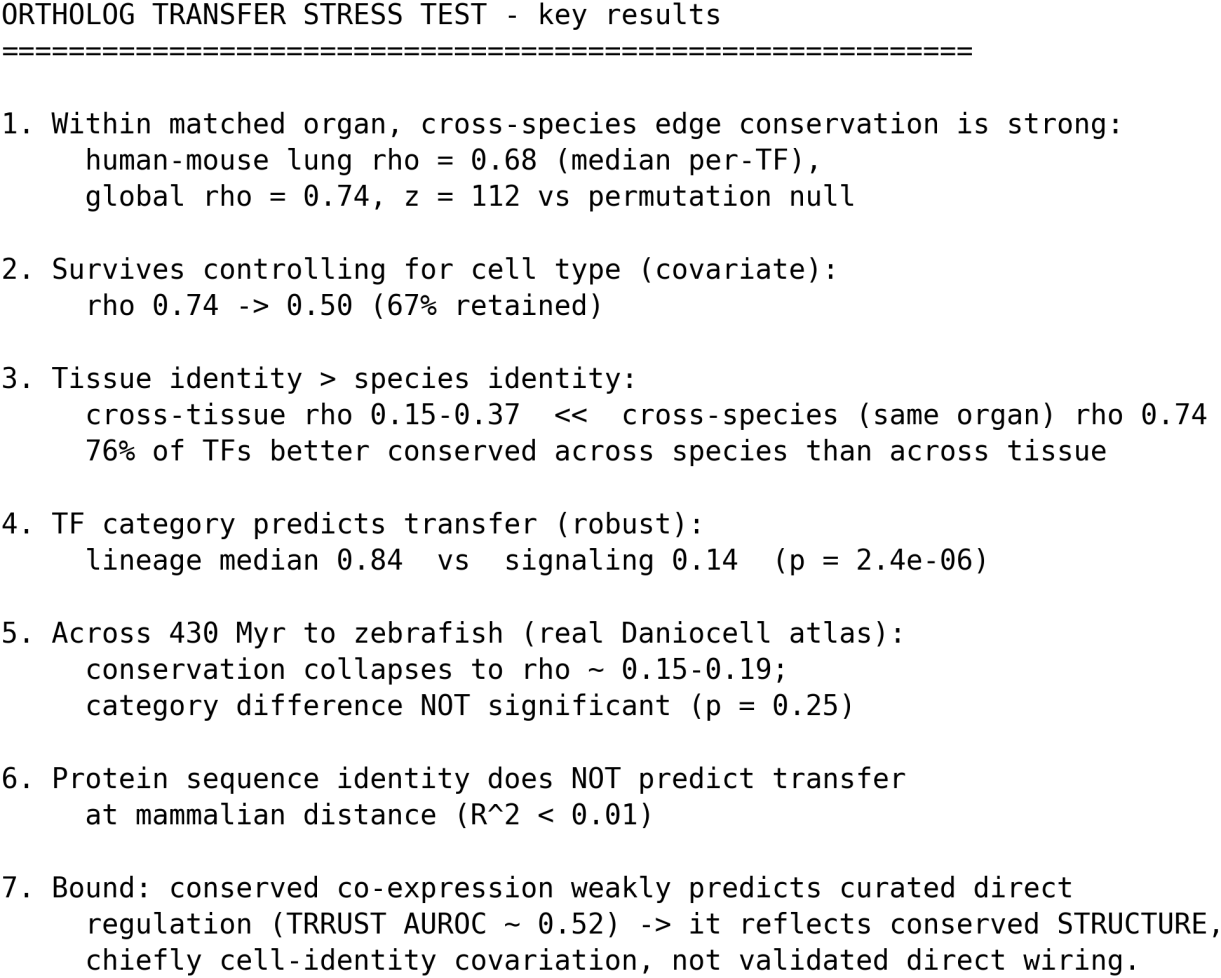
Overall framework. Strong, category-dependent conservation within mammals; primacy of tissue identity; collapse to weak positive agreement by teleost distance; and the bound that conserved co-expression is weakly tied to direct regulation.

In practice: (i) within a matched organ, the strongest co-expression edges of identity-defining TFs can be transferred across mammals with high confidence; (ii) edges of signal-responsive TFs should be treated as species-specific until checked; (iii) tissue matching is more important than species matching—comparing the same organ across species is more meaningful than comparing different organs within a species; and (iv) protein sequence identity is not a useful per-TF prior at mammalian distances. TF functional role, calibrated as above, is the informative prior. The overall framework—strong, category-dependent conservation within mammals, the primacy of tissue identity, the collapse to weak positive agreement by teleost distance, and the weak tie between conserved co-expression and direct regulation—is summarized in Fig 17.

### Limitations

1. **Co-expression, not causation.** Spearman edges capture association, including indirect effects; the weak TRRUST recovery makes this concrete.
2. **One mouse organ.** The cross-species comparison is lung-only because a comparable mouse kidney/immune dataset was unavailable; a full species × tissue design remains future work.
3. **Two divergence times.** With only mammalian and teleost distances, I report conservation at each rather than fitting a decay curve; intermediate species (e.g., chicken) would add resolution.
4. **Zebrafish tissue is not lung.** The zebrafish comparison uses a whole-organism atlas spanning the relevant lineages, the most defensible option given that fish lack a lung; cross-organ effects there are intertwined with evolutionary distance.
5. **Protocol differences.** Human (mixed Smart-seq2/10×) and mouse (Smart-seq2) platforms differ in detection sensitivity.
6. **Sequence proxy.** Whole-protein identity, not domain-restricted identity, was used for the predictive analysis.

## Conclusion

Within a matched organ, co-expression-based regulatory edges transfer strongly across mammals, driven by conserved cell-identity programs, and tissue identity constrains these edges more than species identity does. Transfer is highly TF-dependent: identity factors transfer, signal-responsive factors do not, with a robust category effect within mammals that is no longer resolvable across 430 million years to zebrafish. Conserved co-expression reflects conserved structure—chiefly cell-identity covariation—more than validated direct regulation. These quantitative boundaries indicate when cross-species transfer of regulatory information is safe and when it requires species-specific validation.

## Supporting information

S1 TableThe 61 transcription factors shared between human and mouse lung.For each TF: DNA-binding-domain family, functional category, number of shared edges, cross-species Spearman ρ, sign agreement, Benjamini–Hochberg *q*-value, and the Ensembl (release 116) orthology evidence used in the sequence analysis — ortholog type and whole-protein percent identity for mouse and zebrafish. NA denotes an orthologue that the Ensembl Compara gene-tree pipeline did not resolve.(CSV)
